# Differential cooperation of oncogenes with p53 and Bax to induce apoptosis in rhabdomyosarcoma

**DOI:** 10.1186/1476-4598-5-53

**Published:** 2006-11-02

**Authors:** Alan C Taylor, Katja Schuster, Pamela P McKenzie, Linda C Harris

**Affiliations:** 1 Department of Molecular Pharmacology, Mail Stop 230, St. Jude Children's Research Hospital, Memphis TN 38105, USA; 2 Division of Emergency Medicine, Washington University School of Medicine, St. Louis MO, USA; 3 Simmons Comprehensive Cancer Center, UT South Western Medical Center, Dallas TX, USA

## Abstract

**Background:**

Deregulated expression of oncogenes such as *MYC *and *PAX3-FKHR *often occurs in rhabdomyosarcomas. MYC can enhance cell proliferation and apoptosis under specific conditions, whereas PAX3-FKHR has only been described as anti-apoptotic.

**Results:**

In order to evaluate how MYC and PAX3-FKHR oncogenes influenced p53-mediated apoptosis, rhabdomyosarcoma cells were developed to independently express *MYC *and *PAX3-FKHR *cDNAs. Exogenous wild-type p53 expression in MYC transfected cells resulted in apoptosis, whereas there was only a slight effect in those transfected with PAX3-FKHR. Both oncoproteins induced BAX, but BAX induction alone without expression of wild-type p53 was insufficient to induce apoptosis. Data generated from genetically modified MEFs suggested that expression of all three proteins; MYC, BAX and p53, was required for maximal cell death to occur.

**Conclusion:**

We conclude that cooperation between p53 and oncoproteins to induce apoptosis is dependent upon the specific oncoprotein expressed and that oncogene-mediated induction of BAX is necessary but insufficient to enhance p53-mediated apoptosis. These data demonstrate a novel relationship between MYC and p53-dependent apoptosis, independent of the ability of MYC to induce p53 that may be important in transformed cells other than rhabdomyosarcoma.

## Background

Rhabdomyosarcoma is the most common pediatric soft-tissue sarcoma. The two main subtypes, embryonal and alveolar, are characterized by specific morphologic features and chromosomal translocations. Alveolar rhabdomyosarcomas contain t(2;13) or t(1;13) translocations that generate fusion genes encoding either PAX3 or PAX7 and forkhead (FKHR or FOXO1a) transcription factors [1,2]. The resulting fusion proteins are much stronger transcriptional activators than either PAX3 or PAX7 alone [3]; such increased activity is thought to contribute to the aggressive nature of alveolar rhabdomyosarcoma tumors [4]. PAX3-FKHR expression enhances the proliferation rate and invasiveness of rhabdomyosarcoma tumors [5], and enhances expression of the anti-apoptotic protein BCL-XL [6]. However, tumors with *PAX3-FKHR *often express other deregulated oncogenes [7,8], and *Pax3-FKHR *knock-in mice do not develop tumors [9] suggesting that the oncogenic potential of this fusion protein is weak.

Deregulated expression of members of the *MYC *family of genes is the most common oncogenic change, other than generation of PAX fusion proteins, observed in this tumor type [7,8,10-12]. MYC proteins are involved in the regulation of the cell cycle, proliferation, and apoptosis [13-18]. MYC proteins dimerize with MAX [19] and act as sequence-specific transcriptional activators [20]. By activating the p14^ARF^/p53 pathway, MYC proteins induce apoptosis [21]. Specifically, c-MYC activates ARF, which then binds MDM2; thereby releasing p53 which induces apoptosis [22]. In this manner, cells in which the ARF pathway is functional are protected from the potential transforming effects of MYC protein. However, MYC can induce apoptosis through mechanisms independent of p53 and ARF; for example, MYC can directly induce expression of BAX [23] and ornithine decarboxylase [24], induce release of cytochrome *c *from the mitochondria [25], and play a role in the FAS apoptotic pathway [26]. ARF has also been shown to regulate MYC-mediated apoptosis independent of p53 [27] but to date no relationship between MYC and p53-dependent apoptosis has been described independent of ARF induction.

The goal of the present study was to evaluate how p53-mediated apoptosis is influenced by the expression of two different oncogenes, c-MYC and PAX3-FKHR. We demonstrate that apoptosis can be enhanced in cells that express c-MYC together with wild-type p53 and BAX, but that no similar cooperation exists between PAX3-FKHR and p53 or BAX. In addition, data demonstrate that although c-MYC can induce apoptosis in a p53-independent manner, all three proteins, c-MYC, p53 and BAX are required to induce maximal cell death.

## Results

**Figure 1:**
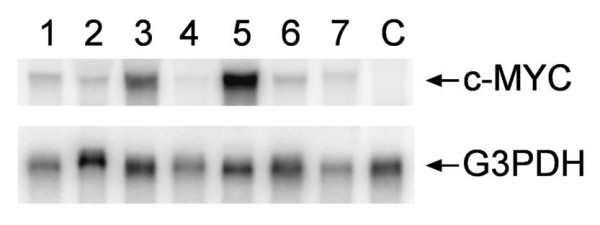
Northern blot analysis of JR1 clones transfected with *c-MYC *cDNA using the *c-MYC *cDNA as a probe. C indicates mRNA isolated from a control clone that had been transfected with the parental vector.

The JR1 rhabdomyosarcoma cell line was chosen for these studies because it was derived from an embryonal tumor; therefore, this line did not contain either the t(1;13) or the t(2;13) translocations that are characteristic of the alveolar subtype. In addition, barely detectable endogenous *c-MYC *mRNA was observed upon Northern blot analysis, and very low MYC-responsive promoter activity was measured (data not shown). Generation of JR1 clones that expressed PAX3-FKHR have been previously described by Shetty et al. [28]. Clones of JR1 cells that expressed c-MYC following transfection and G418 selection were chosen on the basis of mRNA expression (Figure 1). Representative data using c-MYC clone 5 are described.

**Figure 2:**
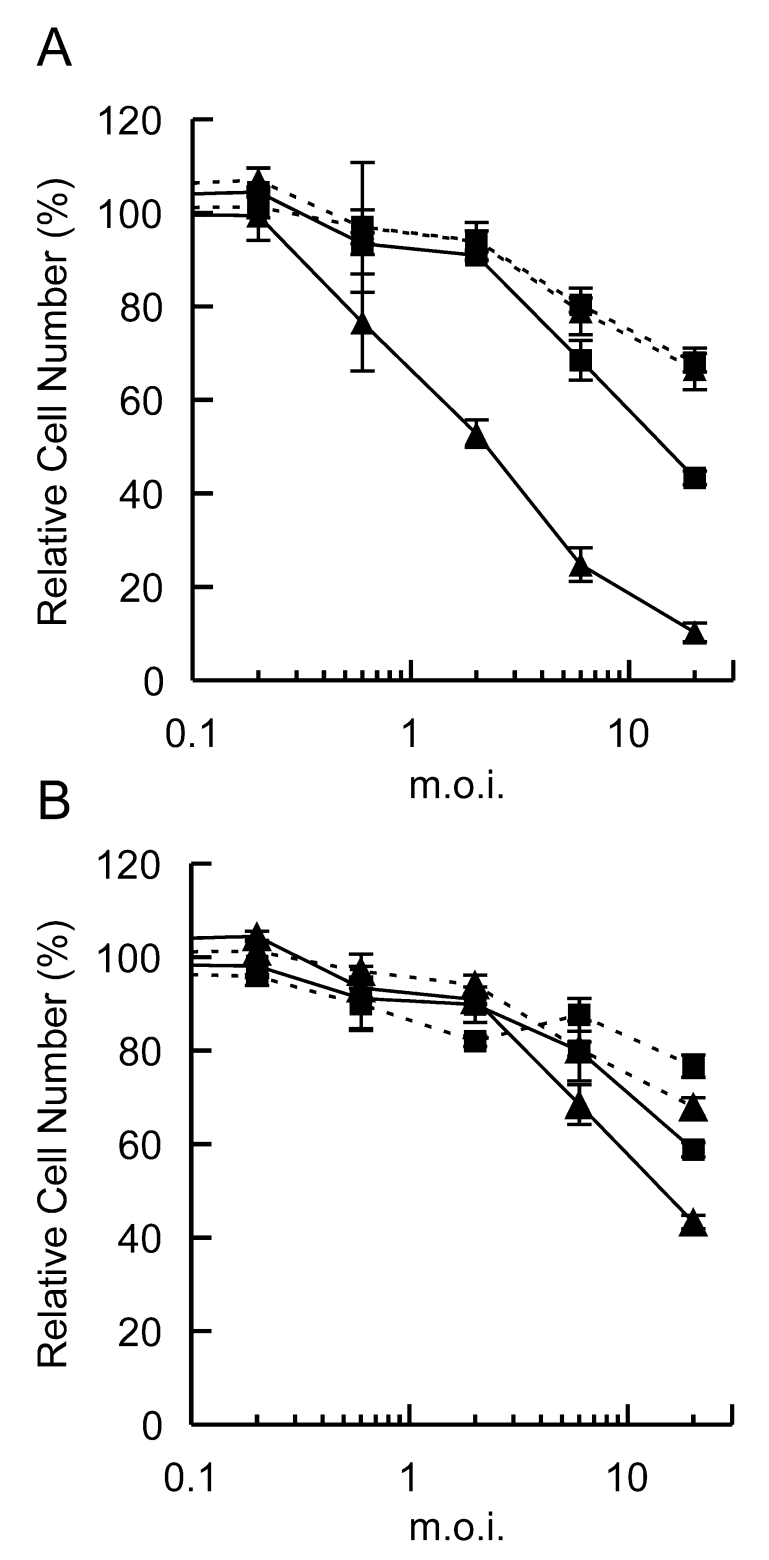
**A**. Cytotoxicity assay of the control (squares) and c-MYC-expressing (triangles) JR1 clones after transduction with Ad-p53 (solid line) and Ad-VC. (dashed line) **B**. Cytotoxicity assay of the control (squares) and PAX3-FKHR-expressing (triangles) JR1 clones after transduction with Ad-p53 (solid line) and Ad-VC (dashed line).

To determine the effect of wild-type p53 expression on survival of JR1 cells that expressed either *c-MYC *or *PAX3-FKHR*, the clones were transduced with increasing concentrations of either Ad-p53 or Ad-VC adenoviral vectors. Data were compared to those obtained from JR1 cells that had been transfected with the parental vector (vector control cells, VC), i.e. those that did not express a transfected oncogene. Expression of exogenous p53 resulted in enhanced cell death compared to when the same cells were transduced with Ad-VC (Figure 2). The c-MYC-expressing cells were as sensitive to Ad-VC transduction as were the control cells (Figure 2A). However, when the c-MYC-expressing cells expressed exogenous wild-type p53 a significant increase in cell death was observed (Figure 2A). These data demonstrated cooperation between p53 and c-MYC in the induction of cell death (Figure 2A). In contrast, the PAX3-FKHR-expressing cells were only slightly more sensitive to Ad-p53 compared to Ad-VC (Figure 2B) demonstrating very little cooperation between p53 and PAX3-FKHR in the induction of cell death.

**Figure 3:**
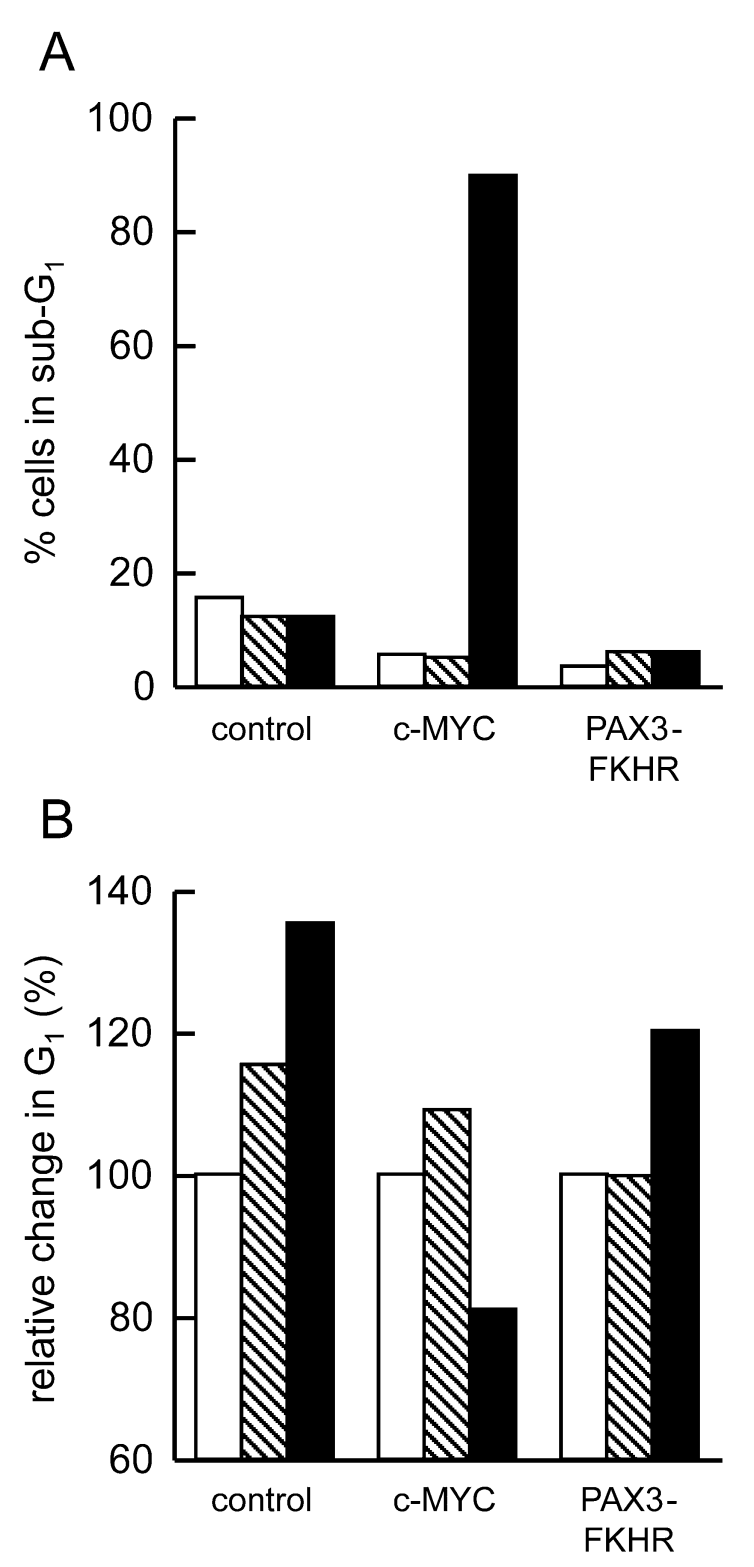
**A**. Representative sub-G_1 _cell-cycle analysis of control cells, c-MYC and PAX3-FKHR-expressing cells. Before analysis, the cells were either untreated (open bars) or exposed to either Ad-VC (m.o.i. 10, shaded bars) or Ad-p53 (m.o.i. 10, solid bars) for 24 h. **B**. Relative change in the proportion of cells in the G_1 _phase of the cell cycle 24 h after exposure to either Ad-VC (m.o.i. 10, shaded bars) or Ad-p53 (m.o.i. 10, solid bars). The values for the untreated cells were set to 100 (open bars). A representative experiment is shown.

To determine whether the observed cell death was due to apoptosis, we carried out cell cycle analysis to evaluate the proportion of cells with a sub-G_1 _DNA content (Figure 3A). An increased proportion of the c-MYC-expressing cells treated with Ad-p53 were in the sub-G_1 _fraction of the cell cycle; therefore, we concluded that these cells were apoptotic. The reduced proportion of cells in the G_1 _phase in these samples also indicated that the observed reduction in cell number (Figure 2A) was from cell death, and not p53-mediated growth arrest in the G_1 _phase of the cell cycle (Figure 3B). Wild-type p53 expression in both the PAX3-FKHR-expressing cells and the control cells resulted in an accumulation of cells in G_1 _(Figure 3B) but not in the sub-G_1 _phase (Figure 3A) of the cell cycle. Therefore, the slight reduction in cell number measured in the cytotoxicity assays upon exogenous p53 expression in both of these cell populations appeared to be due to a reduced growth rate.

**Figure 4:**
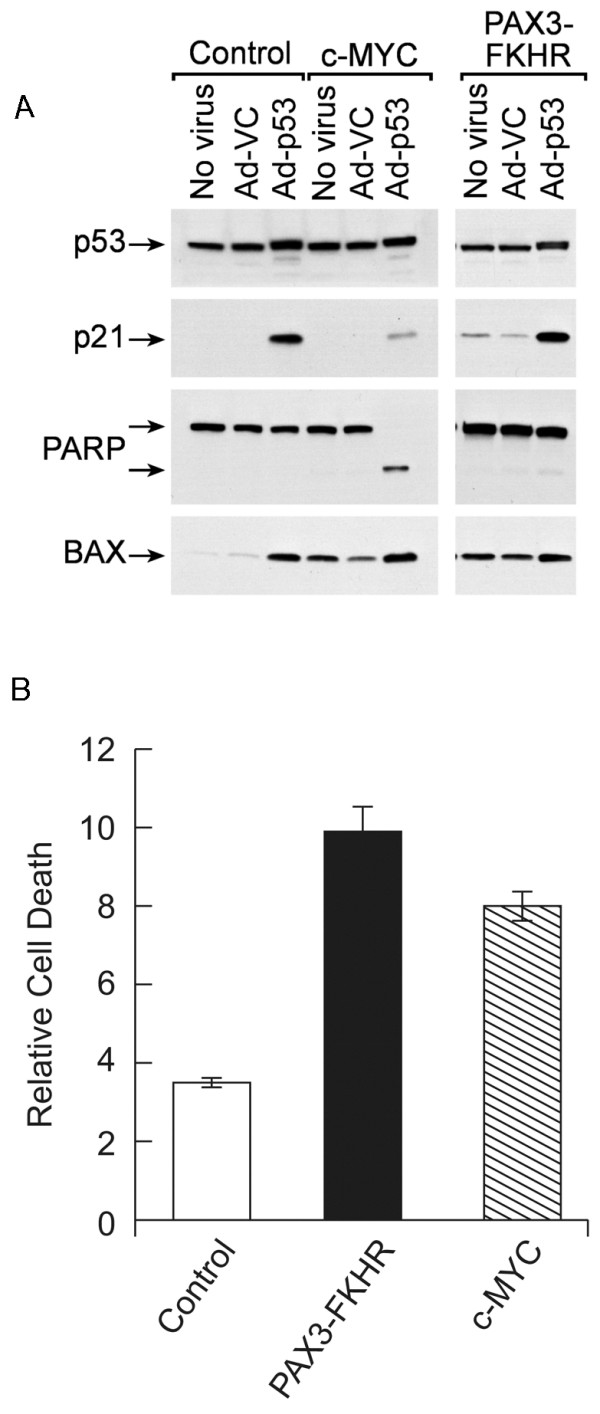
**A**. Western blot analysis of the control, c-MYC- and PAX3-FKHR-expressing cells following exposure to either Ad-VC or Ad-p53 (m.o.i. 10). **B**. Propidium iodide exclusion assay following exposure of the cells to 10 uM doxorubicin for 24 h. Data are presented relative to untreated cells.

As an alternate indicator of apoptosis, Western blot analyses were carried out to evaluate poly (ADP) ribose polymerase (PARP) cleavage in MYC-expressing rhabdomyosarcoma cells. Upon caspase 3 activation, PARP is cleaved into 85 and 25 kDa subunits; the larger of the subunits can be detected by an anti-PARP antibody. Therefore, we indirectly assessed the status of caspase activation by Western analysis to determine whether PARP had been cleaved. PARP cleavage correlated with the accumulation of cells in the sub-G_1 _phase of the cell cycle and the cytotoxicity assay data (Figures 2, 3, 4) demonstrating that cell death was by apoptosis. A slight amount of PARP cleavage was also detected in PAX3-FKHR-expressing cells exposed to Ad-p53 demonstrating that a proportion of these cells were also dying by apoptosis (Figure 4A). These data demonstrate that measurement of PARP cleavage is a more sensitive indicator of apoptosis than the other assays.

To evaluate whether the PAX3-FKHR cells were more resistant to apoptosis induced by exposure to a genotoxic agent, the three cell lines; control, c-MYC and PAX3-FKHR expressing cells were exposed to 10 μM doxorubicin for 24 hours prior to analysis of cell viability by a propidium iodide exclusion assay. Figure 4B demonstrates that the PAX3-FKHR cells were slightly more sensitive to the cytotoxic effects of doxorubicin compared to the MYC expressing cells.

To evaluate whether expression of the pro-apoptotic protein *BAX *was induced upon exogenous expression of either MYC or p53, the same Western membrane used to evaluate PARP cleavage was incubated with anti-BAX antibodies. Elevated BAX expression was observed in all cells that expressed exogenous wild-type p53 or the *c-MYC *or *PAX3-FKHR *oncogenes, including samples in which PARP cleavage was not detected (Figure 4). Therefore, induction of BAX is insufficient to induce apoptosis in the oncogene-expressing cells. Significant apoptosis was only observed in the c-MYC-expressing cells when co-expressed with p53. The same Western membrane was also incubated with antibodies against p53 and p21^Waf1/Cip1^. High-level expression of mutant p53 was observed in all clones, and expression of functional exogenous wild-type p53 was demonstrated by the induction of the p53 target genes, p21 and BAX.

**Figure 5:**
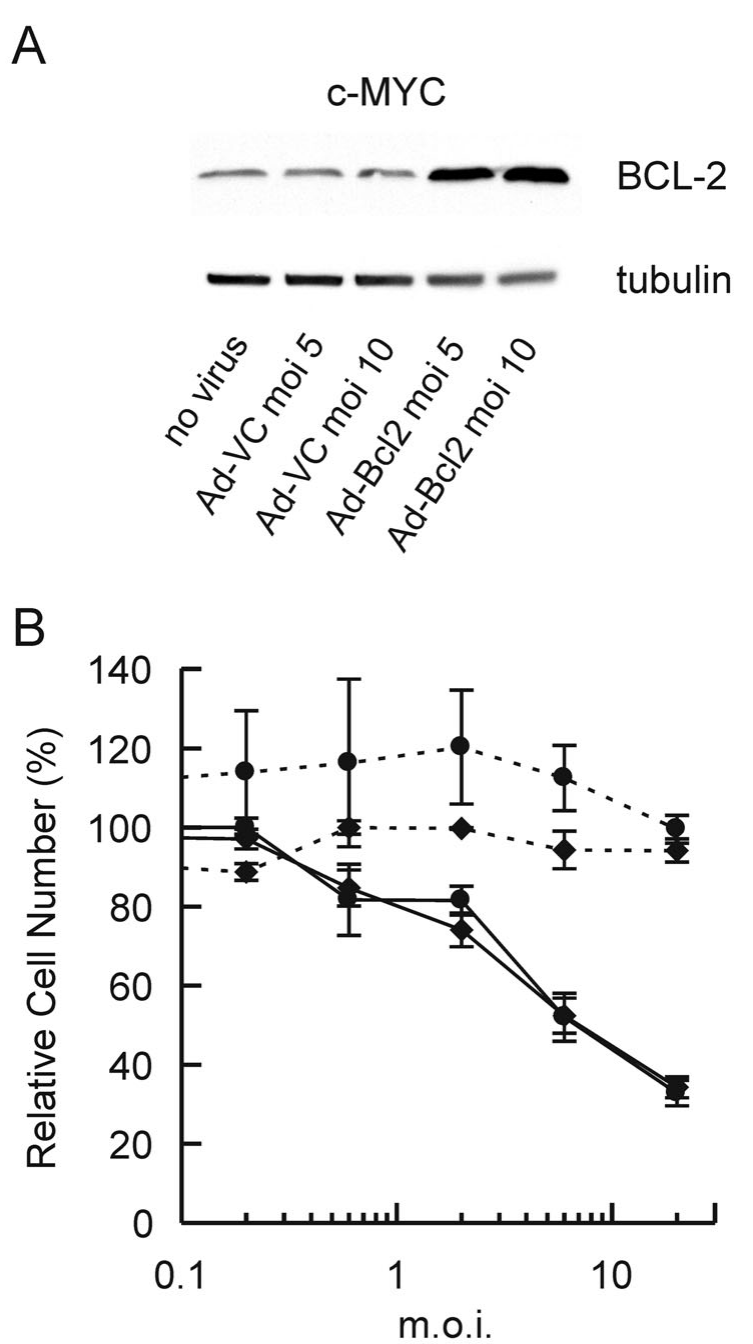
**A**. Western blot analysis of BCL-2 expression in the c-MYC-expressing JR1 cells after transduction with either Ad-BCL2 or Ad-VC. **B**. Cytotoxicity assay of the c-MYC-expressing cells after transduction with different combinations of adenoviral vectors. Cells were pretreated for 24 h with either Ad-VC (m.o.i. 5, circles) or Ad-BCL2 (m.o.i. 5, diamonds) and then exposed to increasing m.o.i.s of either Ad-VC (dashed line) or Ad-p53 (solid line).

MYC-induced apoptosis in certain model systems can be inhibited by BCL-2 [29,30], a protein of the same family that forms heterodimers and inactivates the pro-apoptotic activity of BAX. Therefore, we evaluated the role of BAX in the MYC-induced apoptosis observed in rhabdomyosarcoma cells by analyzing the survival of cells following transduction with Ad-BCL2 in combination with Ad-p53 (Figure 5). Elevated exogenous BCL-2 expression was observed after transduction with Ad-BCL2 at an m.o.i. of 5 and 10 (Figure 5A). However, BCL-2 expression was unable to protect the c-MYC-expressing cells from the apoptotic effects of p53 (Figure 5B). The minimal effect of exogenous BCL-2 expression on MYC-induced apoptosis suggests that BAX plays little or no role in the cooperation between p53 and c-MYC to induce apoptosis of rhabdomyosarcoma cells.

**Figure 6:**
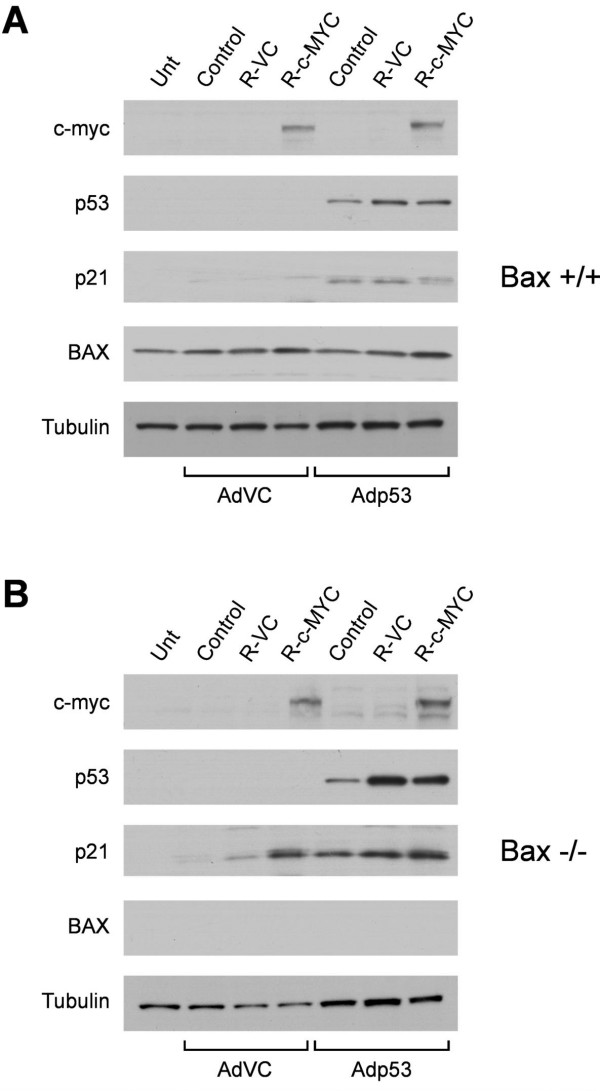
Western blot analysis of Bax+/+ **(A) **and BAX-/- **(B) **MEFs following expression of c-MYC with and without wild-type p53. Unt; untreated cells; R-VC; retrovirus vector control, R-c-MYC; retrovirus c-MYC.

**Figure 7:**
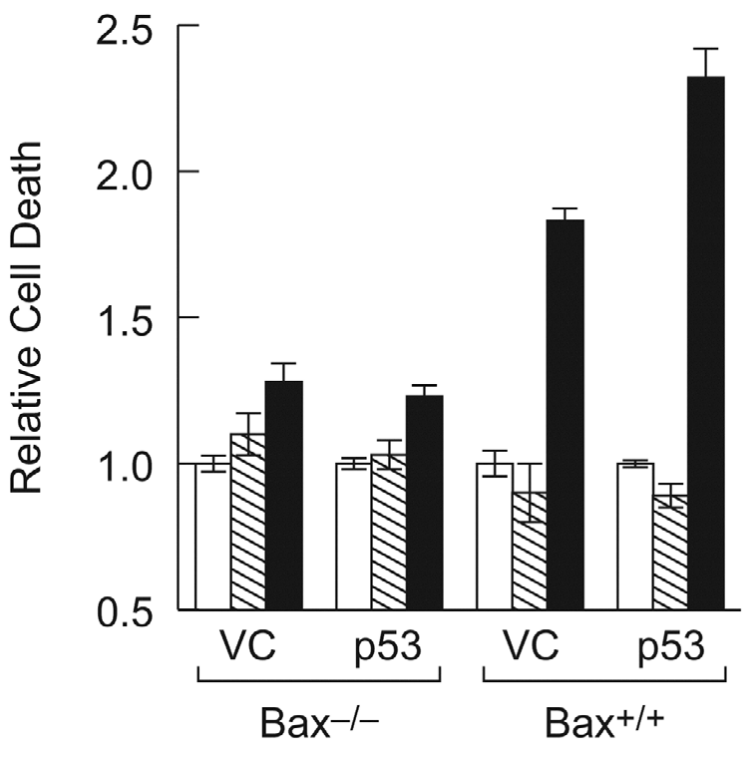
Propidium iodide exclusion cell death assay data for BAX-/- and BAX+/+ MEFs. Open bars represent cells that have not been exposed to any retroviral vector; shaded bars represent retrovirus vector control treated cells and solid bars represent c-MYC retrovirus treated cells. VC represents Ad-VC treated cells and p53 represents Ad-p53 treated cells.

To specifically evaluate cooperation between c-MYC, p53 and BAX in the induction of apoptosis, the relative contribution of each was evaluated in MEFs. Bax-/- and Bax+/+ fibroblasts were used so that the effects of BAX on the induction of apoptosis could be measured. Exogenous wild-type p53 was expressed in these cells by transducing with Ad-p53 (m.o.i. 100), and c-MYC expression was introduced using the retrovirus MSCV-IRES-MYC-ER-GFP, as described in the Methods section. Expression of each of these proteins could be detected by Western analysis (Figure 6). Expression of c-MYC was detectable only in those cells transduced with the MYC retrovirus, and p53 expression was only visible in cells transduced with Ad-p53. Expression of p21 was evaluated as a measure of p53 activity, and although it is detectable in the BAX+/+ cells upon Ad-p53 transduction, it is also visible in the BAX-/- cells upon MYC expression. This result was surprising because MYC has previously been shown to suppress p21 expression [31-33], but it does not influence interpretation of the data generated in this experiment. Measurement of cell death of the BAX-/- and BAX+/+ cells following p53 and c-MYC expression revealed that even though MYC could induce cell death in a p53 independent manner, MYC, p53 and BAX proteins all played a role in the induction of cell death (Figure 7). Maximal cell death was only observed in cells that expressed all three proteins; c-MYC, p53 and BAX.

## Discussion

Rhabdomyosarcoma cells expressing one of the two oncoproteins (c-MYC or PAX3-FKHR) responded in a different manner to exogenous wild-type p53 expression (Figures 2 and 3). PAX3-FKHR has been previously characterized as a relatively weak oncoprotein [9], with an anti-apoptotic phenotype [6]. However in this study no anti-apoptotic effects of PAX3-FKHR expression were observed. Instead PAX3-FKHR weakly enhanced p53-mediated apoptosis.

The oncogenic potential of *c-MYC *is well characterized [16-18], and an apoptotic phenotype has been described [14,34]. Data presented here demonstrate that c-MYC expression alone does not enhance apoptosis of JR1 cells; co-expression with wild-type p53 is required, and this combination resulted in the death of approximately 90% of cells (Figures 2 and 3). A cooperation of wild-type p53 with MYC proteins to induce apoptosis has previously been shown to be ARF-dependent [21] and ARF-independent [35]. ARF binds MDM2 and sequesters it to the nucleolus; thus, MDM2 releases control of p53, and p53 is no longer targeted for degradation by the proteasomal pathway. As a result, p53 protein levels increase [22]. MYC induction of p53 by an ARF-independent mechanism can be mediated through the induction of p53 phosphorylation [35]. However, in the experiments described here p53 and MYC are expressed exogenously and therefore cooperate to induce apoptosis in a manner that is independent of ARF and the ability of MYC to induce p53 expression.

BAX, a proapoptotic member of the BCL-2 family, is a transcriptional target of p53 [36] and MYC [23]. However, Figure 4 shows that elevated BAX expression was observed in the PAX3-FKHR-expressing cells as well as those expressing MYC, demonstrating that BAX can also be induced by PAX3-FKHR. In the control cells that did not express either MYC or PAX3-FKHR, elevated BAX expression was observed only after Ad-p53 transduction. In the cell lines expressing each of the oncoproteins, Ad-p53 transduction only minimally enhanced BAX protein expression above the level induced by the oncogenes. MYC reportedly cooperates with BAX to induce apoptosis [37], and loss of BAX in a transgenic mouse model impairs MYC-induced apoptosis and circumvents the selection for p53 mutations during MYC-mediated lymphomagenesis [38]. However, the results presented in Figures 2, 3, 4 demonstrate that in the JR1 rhabdomyosarcoma cells increased BAX expression induced by either c-MYC or PAX3-FKHR was insufficient to induce apoptosis. The observation that elevated BAX protein together with expression of wild-type p53 did not significantly induce the death of PAX3-FKHR-expressing cells demonstrated that a MYC component is required for the induction of apoptosis by p53 in these cells. We confimed that these results were oncogene specific by demonstrating that the cells expressing either c-MYC or PAX3-FKHR responded in a similar manner when exposed to genotoxic damage (Figure 4B).

Caspase 3 has been shown to be involved in MYC-induced apoptosis [39]. Indeed, our finding that cleavage of the caspase 3 substrate, PARP, was associated with apoptosis of JR1 cells suggested that caspase 3 also played a role in MYC and p53-mediated cell death observed here. Although BCL-2 has been previously shown to inhibit MYC-induced apoptosis [29,30], BCL-2 expression in the MYC-expressing cells did not decrease the proportion of cells that died upon expression of p53 (Figure 5). These data demonstrate that BCL-2 expression only minimally affected the apoptosis of JR1 cells and together with the observation that PAX3-FKHR induced BAX in the same cells with minimal effects on apoptosis suggests that BAX involvement in the MYC and p53-induced cell death is limited. The lower level of p21 induced by p53 in the MYC-expressing cells (Figure 4) is consistent with the published report that MYC downregulates transcription of the p21 promoter [31-33]. MYC suppression of p21 activity has been suggested as a mechanism by which p53 function can be switched from cytostatic to apoptotic [40,41]. This hypothesis is consistent with the results presented in Figure 3, which demonstrate that the proportion of the cell population undergoing apoptosis increased when the G_1 _checkpoint was attenuated. However, p21 expression was elevated in Bax-/- MEFs in response to c-MYC expression (Figure 6). This result does not support previously published data and suggests differences in the response of the rhabdomyosarcoma cells compared to MEFs with respect to MYC expression.

Despite inherent differences in the cell types we evaluated the relative contributions of MYC, p53 and BAX to the induction of apoptosis in MEFs. Only by the use of genetically modified cells, such as Bax-null MEFs, can the effects of each of the three proteins be analyzed independently (Figure 6). In the MEFs, MYC induced cell death independent of wild-type p53. However, maximal cell killing was observed only when all three proteins were expressed together.

The process of immortalization often deregulates cellular apoptotic pathways, for example MYC expression had no effect on survival of JR1 rhabdomyosarcoma cells yet its expression in Bax +/+ MEFs induced cell death. Nevertheless, the cooperation between p53 and MYC to induce apoptosis was observed in both cell types demonstrating that this apoptotic pathway remained intact in rhabdomyosarcoma cells.

## Conclusion

From the data presented here we conclude that the ability of wild-type p53 to induce apoptosis in any given cell type is dependent upon the oncoprotein expressed and that even though different oncoproteins may induce BAX, for example PAX3-FKHR and MYC as shown here, elevated BAX expression is insufficient to induce apoptosis.

## Methods

### Cell lines and transfections

JR1 embryonal rhabdomyosarcoma cells, which contain a p53 Arg248Trp mutation [42], were established at the Institute of Child Health, London, UK [43]. These cells were grown in RPMI-1640 cell culture media supplemented with 10% fetal bovine serum (FBS) in a humidified environment at 37°C and 5% CO_2_, 95% air. The BAX-/- and BAX+/+ mouse embryo fibroblasts (MEFs) were obtained from John Cleveland (St. Jude Children's Research Hospital, Memphis TN) and grown in DMEM media supplemented with 10% FBS, 2% glutamine, 1% non-essential amino acids and 1% β-mercaptoethanol under the same conditions. These cells were grown to at least passage 20 before use in the experiments described in this paper in order to inactivate endogenous p53 activity.

The *c-MYC *cDNA was subcloned into the pIRESneo mammalian expression vector (Clontech, Palo Alto, CA), and the resulting construct was transfected into JR1 cells using the Profectin Mammalian Transfection System (Promega, Madison WI). Individual clones resistant to 200 μg/ml G418 were expanded, and their *c-MYC *mRNA expression was evaluated by Northern blot analysis (Figure 1). Control cells that contained the parental vector were developed by transfection with the pIRESneo plasmid that did not contain the *c-MYC *cDNA. Transcriptional activity of c-MYC in the transfected clones was analyzed using a MYC-responsive promoter reporter plasmid [44] and a dual luciferase assay (Promega). We have previously described the generation and characterization of JR1 cells expressing the *PAX3-FKHR *cDNA [28]. RT-PCR showed that these cells express *PAX3-FKHR*, and luciferase assays of cells that had been transfected with a PAX-responsive luciferase reporter plasmid demonstrated that PAX transcriptional activity in the transfected clones was increased [28].

### Viral vectors and cell transduction

Ad-p53 (Av1p53) was provided by Genetic Therapy Inc. (a Novartis Company, Gaithersburg MD) [45]. Ad-VC and Ad-Bcl2 were provided by Dr. Janet Houghton (St. Jude). Cells were transduced with adenoviral vectors at the multiplicity of infection (m.o.i.) as described in the Results. Retroviral plasmids; MSCV-IRES-MYC-ER-GFP and MSCV-IRES-GFP, were obtained from John Cleveland (St. Jude) and have previously been described [21]. These plasmids were independently transiently transfected with an ecotropic helper retroviral plasmid into 293T packaging cells in order to generate retroviral particles. Retroviral supernatant was harvested from the 293T cells at 24 and 48 h following transfection. This supernatant was filtered and added to MEFs (at a dilution of 1:2) together with hexadimethrinebromide (Polybrene, Sigma) at a final concentration of 1 μg/ml. After 24 h 4-hydroxytamoxifen (Sigma) was added to a final concentration of 1 μM to induce MYC expression. Cells were harvested after a further 24 h for Western and PI exclusion analyses.

### Cytotoxicity assay and cell cycle analysis

Cells were plated in triplicate at a density of 1 × 10^5 ^per well in 6-well plates. After a 24-h period of attachment, cells were exposed to adenoviral vectors whose m.o.i. ranged from 0.2 to 20. The total number of cells in each well was counted after the untreated cells had doubled 3 times. Data are presented as a percentage of untreated cells.

Cell cycle analysis was carried out on cells transduced with virus (m.o.i. = 10) for 24 h. Cells were suspended at a concentration of 1 × 10^6^/ml in a solution of propidium iodide, and their DNA content was analyzed as previously described [46]. Propidium iodide cell exclusion assays were also carried out as a measure of cell death. Pelleted cells were resuspended in the propidium iodide solution used for DNA content analysis that did not contain any Triton X-100, and analyzed by flow cytometry.

### Northern and Western blot analyses

Northern blot analysis was conducted as described by Sambrook et al. [47]. Cells for Western blot analysis were transduced for 24 h with the adenoviral vectors at an m.o.i. of 10. Cell extracts were prepared, and Western blot analyses were performed as previously described [46]. The p53 antibody (DO1-HRP) and the antibodies against BAX, BCL-2, c-MYC and p21 were obtained from Santa Cruz Biotechnology (Santa Cruz, CA). The poly (ADP ribose) polymerase (PARP) antibody was purchased from PharMingen (San Diego, CA), and the β-tubulin antibody was obtained from ICN Biomedicals, Inc (Aurora, OH).

## Abbreviations

MEF, mouse embryo fibroblast; m.o.i., multiplicity of infection; PARP, poly (ADP ribose) polymerase; VC, vector control.

## Competing interests

The author(s) declare that they have no competing interests.

## Authors' contributions

ACT, KS and PPM were all involved in data acquisition. LCH designed and coordinated the study, and also drafted the manuscript. All authors were involved in data interpretation, and critically read and approved the final manuscript.
